# Duodenal neuroendocrine tumours masquerading as peptic duodenitis, crushed lymphoid aggregates and prominent Brunner glands: some diagnostic pitfalls

**DOI:** 10.1111/his.15463

**Published:** 2025-04-27

**Authors:** Lena Hasson, Dipti M Karamchandani

**Affiliations:** ^1^ Department of Pathology UT Southwestern Medical Center Dallas TX USA

**Abbreviations**
GI: gastrointestinal
INSM1: insulinoma‐associated protein 1
NET: neuroendocrine tumour

Duodenal neuroendocrine tumours (NETs) are rare tumours, accounting for approximately 2.8% of all NETs and 4% of all gastrointestinal (GI)‐NETs, but with an increasing incidence probably secondary to advances in endoscopic imaging and earlier detection.^1^, ^2^, ^3^ We present here two cases of duodenal NETs masquerading as benign aetiologies, with discussion of associated diagnostic pitfalls.

The first patient was a 64‐year‐old woman with metastatic breast carcinoma on therapy, who underwent upper GI endoscopy for anaemia and post‐prandial diarrhoea that revealed a single 6‐mm submucosal nodule with a localised distribution in the duodenal bulb. Histopathological examination revealed two fragments of duodenal mucosa with reactive epithelial changes, including non‐specific villous blunting and gastric metaplasia, consistent with peptic duodenitis. Additionally, one of the two fragments showed crushed blue cells in the submucosa, mimicking crushed lymphoid cells (Figure 1A–D). Given the crush artefact, it was difficult to discern the morphology of these cells on haematoxylin and eosin‐stained slides. The second patient was a 64‐year‐old woman presenting with abdominal pain, and an upper GI endoscopy showed a single 6‐mm mucosal nodule with localised distribution in the duodenal bulb. Histopathological examination showed duodenal mucosa with gastric metaplasia and non‐specific villous blunting, consistent with peptic duodenitis. Additionally, a higher‐powered examination showed some glands interspersed within non‐neoplastic duodenal crypts remarkably similar to Brunner glands, but were identified in the superficial mucosa, a location unusual for Brunner glands. These Brunner‐like glands showed abundant frothy lightly eosinophilic cytoplasm and basally located bland nuclei (Figure 2A–D). The presence of peptic duodenitis in both cases could have explained the ‘nodular’ appearance seen on endoscopy, in addition to the crushed lymphoid‐like cells in the first case. However, given the presence of these crushed blue ‘lymphoid‐like’ cells in the submucosa (in the first case) and the aberrant location of the ‘Brunner‐like’ glands (in the second case), ancillary stains were performed. The lesional cells in both cases were positive for synaptophysin, chromogranin A, insulinoma‐associated protein 1 (INSM1) and cytokeratin (AE1/AE3), supporting the diagnosis of a NET. The Ki67 index was assessed to be approximately 2% in both cases on this limited lesional material, favouring a grade 1 NET. Clinical management for the first patient consisted of close observation and repeated endoscopy in 6 months, given the ongoing chemotherapy for her breast cancer. The second patient underwent an endoscopic resection of the nodule, which was consistent with well‐differentiated neuroendocrine tumour (residual 0.4 cm), with a Ki67 labelling index of 4.3%, consistent with a grade 2 tumour. Staging scans confirmed no residual disease, and the plan was for active endoscopic surveillance.

**Figure 1:**
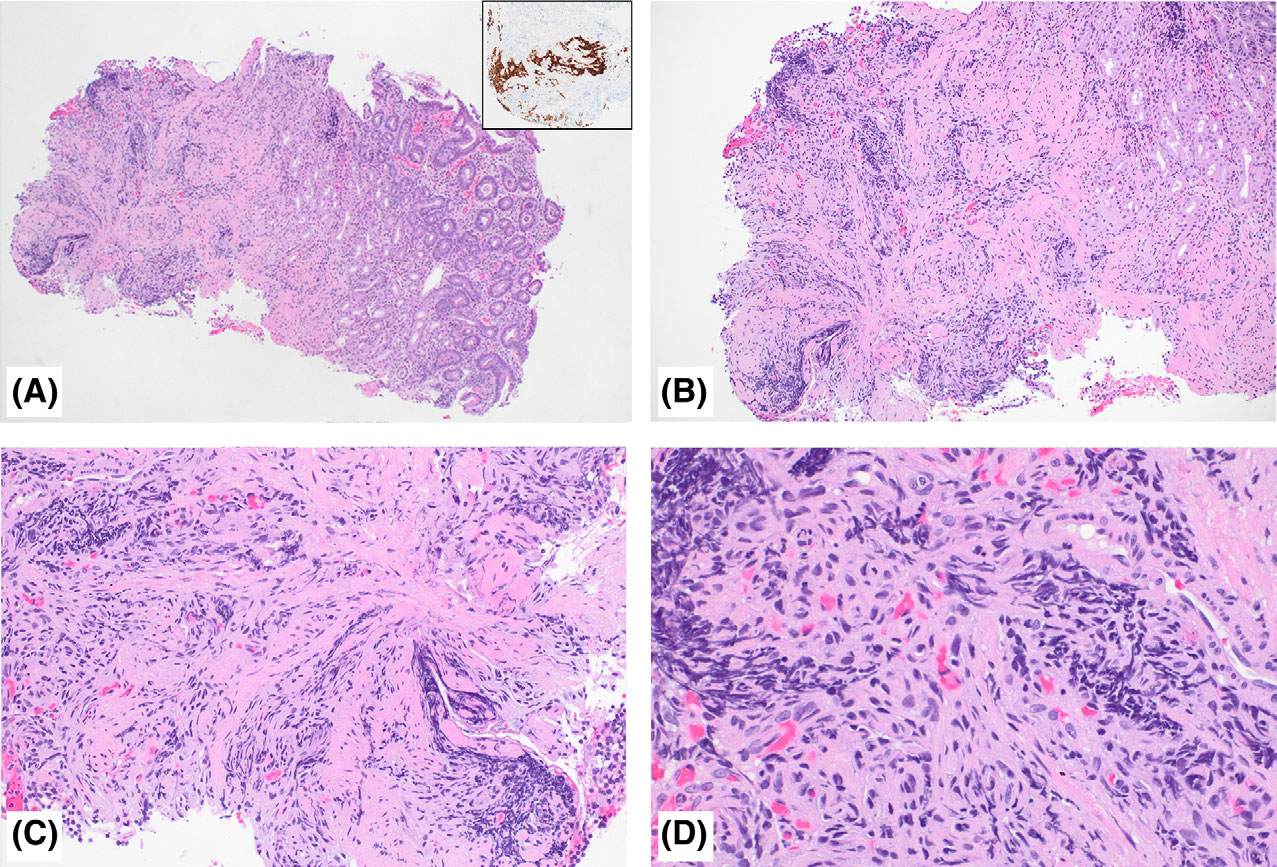
Duodenal neuroendocrine tumour (NET) mimicking crushed lymphoid cells in the submucosa. **A**, Low‐power photomicrograph showing biopsy fragment with peptic duodenitis and with crushed blue cells in the submucosa resembling crushed lymphoid cells. Inset highlights insulinoma‐associated protein 1 (INSM1) immunostain positivity in the crushed cells, supporting a diagnosis of NET. **B–D**, Medium‐ and higher‐power photomicrographs showing the crushed blue cells in the submucosa. On review, some of the cells may raise a possibility of NET; however the histomorphological features are difficult to discern because of the crush artefact and these can be misdiagnosed as crushed lymphoid cells due to the subtlety of these findings.

**Figure 2:**
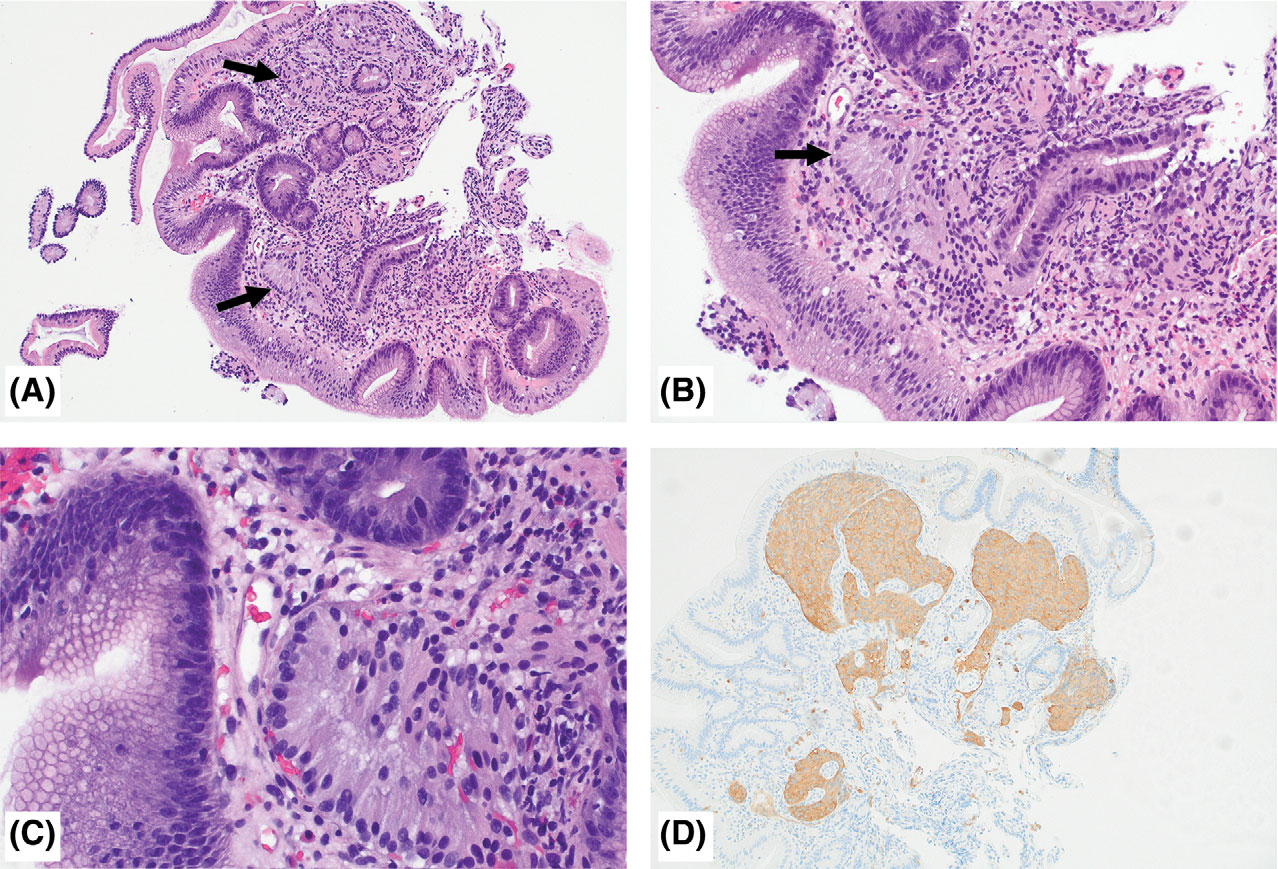
Duodenal neuroendocrine tumour (NET) mimicking Brunner glands. **A,B**, Low‐ and medium‐power photomicrograph showing prominent non‐specific villous blunting and gastric metaplasia, consistent with peptic duodenitis. Glands that resemble Brunner glands (highlighted by arrows) are seen; however, these are seen within the duodenal crypts in the superficial mucosa, an aberrant location for Brunner glands. **C**, Higher‐power photomicrograph highlighting the abundant frothy lightly eosinophilic cytoplasm and basally orientated nuclei in these glands, resembling Brunner glands. **D**, Immunostain for synaptophysin highlights positivity in these glands, supporting a diagnosis of NET.

With a progressive increase in endoscopy procedures, pathologists are bound to see an increased number of duodenal NETs in their routine clinical practice. The majority of these are non‐functional; hence, many are incidentally discovered on endoscopy. The existing data describe them endoscopically as small, sessile erythematous or pale lesions or submucosal hemispherical or flatly elevated lesions that can be seen in the duodenal cap, bulb, first or second part of the duodenum.^1^, ^4^ However, in routine clinical practice, we have seen them as lumped together and often described as a mucosal or submucosal nodule. In addition to NETs, a ‘nodular’ endoscopic appearance can have varied histological differentials, including benign aetiologies such as Brunner gland hyperplasia, gastric heterotopia, peptic duodenitis and lymphoid hyperplasia, among others. Once diagnosed there is very limited evidence for a wait‐and‐watch policy, and it is generally recommended that the tumour be completely excised using endoscopic resection or even local resection, if endoscopic therapy is high risk or unlikely to be curative.^1^, ^4^ Treatment is based on tumour size, location, histological grade, stage and tumour type. Given that metastases to lymph nodes and liver from duodenal NET are known to occur and even patients with tumour size ≤ 1 cm have a 40% chance of nodal metastases, an early and accurate diagnosis is essential.^4^, ^5^ The pathological assessment and grading of duodenal NETs is akin to NETs at other GI sites.^6^

To summarise, pathologists should always consider a possibility of NET when encountered with a duodenal mucosal or submucosal nodule, despite the presence of other confounding aetiologies, such as peptic duodenitis, that can account in part for the ‘nodular’ appearance. Pathologists need to be cognisant that NETs in duodenum can show crush artefact with crushed tumour cells mimicking crushed lymphoid cells, a diagnostic pitfall. Another pitfall is that these tumour cells can also mimic Brunner glands; however, the presence of Brunner‐like glands in the superficial mucosa or showing an infiltrative appearing pattern should alert pathologists to consider the possibility of NET.

## Conflicts of interest

The authors have no conflicts of interest to declare.

## Data Availability

Data sharing is not applicable to this article, as no data sets were generated or analysed during the current study.
